# Chimeric Antigen Receptor T-Cell Therapy Is Associated with Low Absolute Rates of Orofacial Adverse Events and Significantly Less When Compared to Hemopoietic Stem Cell Therapy

**DOI:** 10.3390/dj14090549

**Published:** 2026-09-01

**Authors:** Stella O. Oyewole, Emma Butler, Sagun D. Goyal, Adepitan A. Owosho

**Affiliations:** 1 College of Medicine, University of Cincinnati, Cincinnati, OH 45267, USA; 2 Oral Medicine, Dental Oncology and Urgent Care Unit, Missouri School of Dentistry and Oral Health, A.T. Still University, 1500 Park Avenue, St. Louis, MO 63104, USA; 3 Bone Marrow Transplant & Cellular Therapy Unit, Department of Medicine, School of Medicine, Saint Louis University, St. Louis, MO 63104, USA; 4 Robert Ebert and Greg Stubblefield Head and Neck Tumor Center, Siteman Cancer Center, St. Louis, MO 63108, USA

**Keywords:** CAR T-cell therapy, treatment-related adverse events, oral adverse events, oral mucositis, stomatitis

## Abstract

**Background/Objectives:** Chimeric antigen receptor T-cell (CAR-T) therapy has been approved for the management of relapsed and refractory hematologic malignancies, transforming the oncologic landscape and producing durable remissions in patient populations with limited alternatives. The systemic adverse events of CAR-T are well characterized; however, the orofacial adverse events have not been well described. The objective of this study is to leverage a large, de-identified, real-world dataset to (1) estimate the prevalence of orofacial adverse events following CAR-T, (2) compare event rates with the general population, and (3) directly contrast the orofacial toxicity burden of CAR-T with that observed after hemopoietic stem cell transplant (HSCT). **Methods:** We performed a retrospective cohort study using de-identified electronic health record data from TriNetX. CAR-T and HSCT cohorts were identified via RxNorm and procedure codes; a non-exposed control cohort was included. Patients with prior orofacial conditions or confounding therapies were excluded. New adverse orofacial events within one year were identified by International Classification of Diseases, 10th Revision (ICD-10) codes. Cohorts were 1:1 propensity-matched by age and sex; associations were estimated as odds ratios with two-sided 95% CIs. **Results:** In 1142 CAR-T recipients (mean age of 62), gastroesophageal reflux disease (GERD) was most frequent (5.18%). Oral mucosal events included stomatitis in 1.52%, mucositis in 1.31%, and lichenoid reactions in 1.03%. Dysphagia occurred in 1.8% and oral candidiasis in 1.6%. Several severe oral conditions were absent. Compared with the general population (CART vs. general population): mucositis—[12/995 vs. 0/1112] (OR 28.3)—and stomatitis—[16/987 vs. 0/1119] (OR 38)—risks were significantly increased, while CAR-T patients had significant lower risks of mucosal complications than HSCT recipients in this analysis (CART vs. HSCT): mucositis—[1.21% vs. 3.72%] (OR 0.316) and stomatitis—[1.42% vs. 3.91%] (OR 0.354). **Conclusions:** CAR-T therapy carries a distinct and generally lower orofacial toxicity burden than HSCT, but targeted dental assessment and prospective surveillance remain important to optimize supportive care for cellular therapy recipients.

## 1. Introduction

Chimeric antigen receptor T-cell (CAR-T) therapy has transformed the management of relapsed and refractory hematologic malignancies, producing durable remissions in patient populations with limited alternatives [1,2,3,4,5,6,7]. Several CAR T-cell products targeting Cluster of Differentiation 19 and B-cell maturation antigen (BCMA) have received FDA approval for hematologic malignancies. CD19-directed therapies include tisagenlecleucel (Kymriah^®^) for acute lymphoblastic leukemia (ALL) and diffuse large B-cell lymphoma (DLBCL); axicabtagene ciloleucel (Yescarta^®^) for DLBCL, primary mediastinal LBCL, and high-grade B-cell lymphoma; brexucabtagene autoleucel (Tecartus^®^) for mantle cell lymphoma and adult B-cell ALL; lisocabtagene maraleucel (Breyanzi^®^) for DLBCL, follicular lymphoma, and primary mediastinal LBCL; and obecabtagene autoleucel (Aucatzyl^®^) for relapsed/refractory adult B-cell ALL. BCMA-targeted agents include ciltacabtagene autoleucel (Carvykti^®^) and idecabtagene vicleucel (Abecma^®^), both approved for relapsed/refractory multiple myeloma. The dominant and well-described toxicities of CAR-T are systemic immune-mediated syndromes, cytokine release syndrome (CRS), immune effector cell-associated neurotoxicity syndrome (ICANS), and immune effector cell-associated hematotoxicity (ICAHT), which have driven the development of standardized recognition and management algorithms in clinical practice [8,9,10,11,12,13]. CRS typically presents within days of infusion with fever, hypotension, and respiratory compromise driven by excessive cytokine release (IL-6, TNF-α and IFN-γ) with an incidence of 49–95% reported in clinical trials [8]; ICANS manifests with a spectrum of neurologic deficits (tremors, dysgraphia, expressive aphasia, reduced consciousness, seizures, and cerebral edema) linked to endothelial activation and blood–brain barrier disruption; and ICAHT produces biphasic cytopenias related to conditioning regimen and CAR-T-mediated marrow suppression.

By contrast, reports of orofacial adverse events following CAR-T are sparse, consisting mainly of a small case series [14]. Pharmacovigilance analyses and recent reviews highlight a broad spectrum of CAR-T-related adverse events, but little to no oral events are reported relative to neurologic and systemic toxicities [8,15,16,17]. Understanding CAR-T oral toxicity is important because orofacial complications can substantially impair nutrition, oral function, and quality of life, and may complicate oncologic care. In contrast to CAR-T, hematopoietic stem cell transplantation (HSCT), whether allogeneic or autologous, is well known to cause frequent and often severe oral complications, including high rates of acute oral mucositis during conditioning [18,19]. Chronic oral graft-versus-host disease (cGVHD), which occurs mainly after allogeneic HSCT, manifests as mucosal ulceration, lichenoid changes, dysgeusia, and salivary dysfunction [20,21]. These HSCT-related oral sequelae are associated with prolonged morbidity and increased healthcare utilization. CAR-T-specific oral adverse event data from registries or pharmacovigilance databases are not available and comparisons with HSCT are not available by differing patient populations, conditioning regimens, and surveillance intensity. A recent retrospective study describing oral manifestations in CAR-T recipients reported manageable rates of xerostomia and neuropathic and infectious findings, underscoring the need for systematic surveillance and standardized reporting [14]. A recent review of oral toxicities associated with CAR-T therapy proposed several mechanistic pathways underlying possible oral manifestations. Oral ulcerations may arise secondary to CRS, particularly in the setting of elevated IL-6. Infectious oral conditions are thought to reflect treatment-related immunosuppression and cytopenia. Xerostomia has been linked to off-target antigen recognition, while periodontal disease may develop as a consequence of therapy-induced alterations in the oral microbiome [22].

Given these gaps, comparative analyses that quantify both absolute incidence and relative risk of orofacial events after CAR-T versus HSCT are needed to inform pre-treatment dental evaluation, peri-therapy oral care pathways, and survivorship planning. This study addresses that need by leveraging a large, de-identified, real-world dataset to (1) estimate the prevalence of orofacial adverse events following CAR-T, (2) compare event rates with the general population, and (3) directly contrast the orofacial toxicity burden of CAR-T with that observed after HSCT. The findings aim to guide clinicians on risk stratification, dental referral practices, and targeted monitoring for patients undergoing cellular therapies.

## 2. Methods

### 2.1. Data Source

Data for this retrospective cohort study were obtained from the TriNetX Research Network (TRN), a global federated health research platform that provides access to de-identified electronic health record (EHR) data from 111 healthcare organizations. The TriNetX platform enables standardized cohort identification and outcome analyses using harmonized clinical data across participating healthcare systems.

### 2.2. Study Population and Cohort Definition

Patients who received chimeric antigen receptor T-cell (CAR-T) therapy were identified using medication records mapped to RxNorm codes corresponding to all current FDA-approved CAR-T therapy products (Table 1). Patients who underwent hematopoietic stem cell transplantation (HSCT) were similarly identified using relevant diagnostic and procedure codes (Table 1). The primary comparison cohort consisted of patients with no documented history of CAR T-cell therapy. Similar inclusion and exclusion criteria were applied across cohorts to improve comparability between groups.

Patients with pre-existing orofacial conditions prior to cohort entry were excluded to ensure that identified outcomes represented new-onset events following CAR T-cell therapy. To minimize potential confounding, patients receiving treatments or medications known to independently influence oral mucosal health, salivary function, and neurological status were excluded. These exclusions included classic chemotherapeutic agents (cisplatin, doxorubicin, methotrexate, and cyclophosphamide), monoclonal antibodies (including rituximab and trastuzumab), immune checkpoint inhibitors (including pembrolizumab, nivolumab, and ipilimumab), hormonal therapies, long-term immunosuppressive therapy, and a history of head and neck radiotherapy.

### 2.3. Study Timeline

To maximize the available sample size within the TriNetX database, no restrictions were placed on the calendar period of cohort entry. The index date was defined as the first documented receipt of CAR-T therapy. Patients with pre-existing orofacial adverse conditions, defined as those who had experienced any of the orofacial adverse events evaluated in our study before receiving CAR-T therapy or undergoing HSCT, were excluded to ensure that exposure preceded outcome development.

### 2.4. Outcome Measures

The primary outcome was the development of new-onset orofacial adverse events within a year following CAR-T therapy. Outcomes were categorized into disturbances of salivary secretion, oral mucosal changes, orofacial neuropathies, medication-related osteonecrosis of the jaw (MRONJ), dental caries, gastroesophageal reflux disease (GERD), dental erosion, halitosis, chronic pharyngitis, graft-versus-host disease (GvHD), and oral hyperpigmentation. GERD was evaluated because of its well-established association with clinically relevant oral manifestations, including dental erosion, oral mucosal irritation, and burning mouth symptoms, which may be particularly relevant in patients undergoing CAR-T therapy or HSCT. Outcomes were identified using ICD-10 diagnostic codes as follows: Disturbances of salivary secretion: K11.7, R68.2, and M35.0. Oral mucosal conditions: lichenoid reactions: T78.40XA, L44.2, and L27.0; oral mucositis: K12.30, K12.3, K12.39, and K12.31; stomatitis/necrotizing ulcerative stomatitis: K12.1, K12, K12.0, and A69.0; oral leukoplakia: K13.29, K13.2, K13, and K13.3; erythema multiforme/Stevens–Johnson syndrome: L51.9, L51, and L51.8; oral lichen planus: L43; oral candidiasis: B37.0; gingival hyperplasia: K06.1. Orofacial neuropathies: dysgeusia: R43.2, R43.0, and R43.9; aphagia: R13.0; dysphagia: R13.1, R13.10, R13.12, R13.14, R13.13, and R13.11; neuralgia/glossodynia: K14.6 and T28.0, MRONJ: M87.180. Additional hard oral tissues and gastrointestinal outcomes: dental caries: K02, K02.9, K02.6, K02.5, K02.63, K02.62, K02.52, K02.3, K02.53, K02.61, K02.7, and K02.51; GERD: K12.9, K12.0, K12.00, and K12.01; dental erosion: K03.2; halitosis: R19.6; chronic pharyngitis: J31.2, J31.1, and R07.0; GvHD: D89.813, D89.811, D89.810, D89.812, and D89.81; and oral hyperpigmentation: L81.4 and L81.0.

### 2.5. Statistical Analysis

The CAR-T therapy cohort and control cohort were matched using 1:1 propensity score matching based on age and sex. Propensity score matching was performed using the built-in propensity score matching tool within the TriNetX platform. The TriNetX platform provides post-matching assessment of cohort balance using SMDs. All SMD values for the evaluated covariates were <0.1, indicating adequate balance between the CAR-T therapy and HSCT cohorts after matching. The specific matching algorithm and caliper width parameters are determined by the TriNetX platform and are not user-configurable or available within the analytic interface. Associations between CAR-T therapy exposure and orofacial adverse outcomes were assessed using odds ratios (ORs) with 95% confidence intervals (CIs). Additionally, the occurrence of orofacial adverse outcomes was compared between patients receiving CAR-T therapy and those receiving HSCT to evaluate differences in risk between these treatment groups.

All analyses were performed on 18 June 2026 within the secure TriNetX LIVE™ environment. Only aggregated and de-identified results were accessible to investigators, and no protected health information was available. In accordance with TriNetX privacy policies, patient counts ≤ 10 were automatically rounded to preserve confidentiality. This study was reviewed by the A.T. Still University IRB and was found to be in the exempt category according to 45CFR46.104 (d)(4)(i). The IRB number for this protocol is AO20250707-001. Statistical significance was defined as a two-sided *p*-value <0.05.

## 3. Results

### 3.1. Prevalence of Orofacial Adverse Events in CAR-T Recipients

In a cohort of 1142 patients who received CAR-T therapy (mean age of 62± 17 years), and after excluding individuals with prior related outcomes, the most frequently recorded orofacial event was gastroesophageal reflux disease (GERD) (5.18%, *n* = 45). Among oral mucosal disorders, stomatitis occurred in 1.52% (*n* = 15), oral mucositis in 1.31% (*n* = 13), and oral lichenoid drug reactions in 1.03% (*n* = 11). Dysphagia was reported in 1.8% (*n* = 19), and infectious oral conditions such as oral candidiasis were recorded in 1.6% (*n* = 17). The following conditions were not observed in the CAR-T cohort: erythema multiforme, Stevens–Johnson syndrome, lichen planus, oral hairy leukoplakia, gingival hyperplasia, aphagia, dental erosion, and MRONJ. Counts for xerostomia, dysgeusia, neuralgia, paresthesia, glossodynia, and halitosis were suppressed by the database policy (counts < 10) and therefore are not reported (see Table 2 for full prevalence data).

### 3.2. Association of CAR-T Therapy with Orofacial Adverse Events Versus the General Population

**Figure 1:**
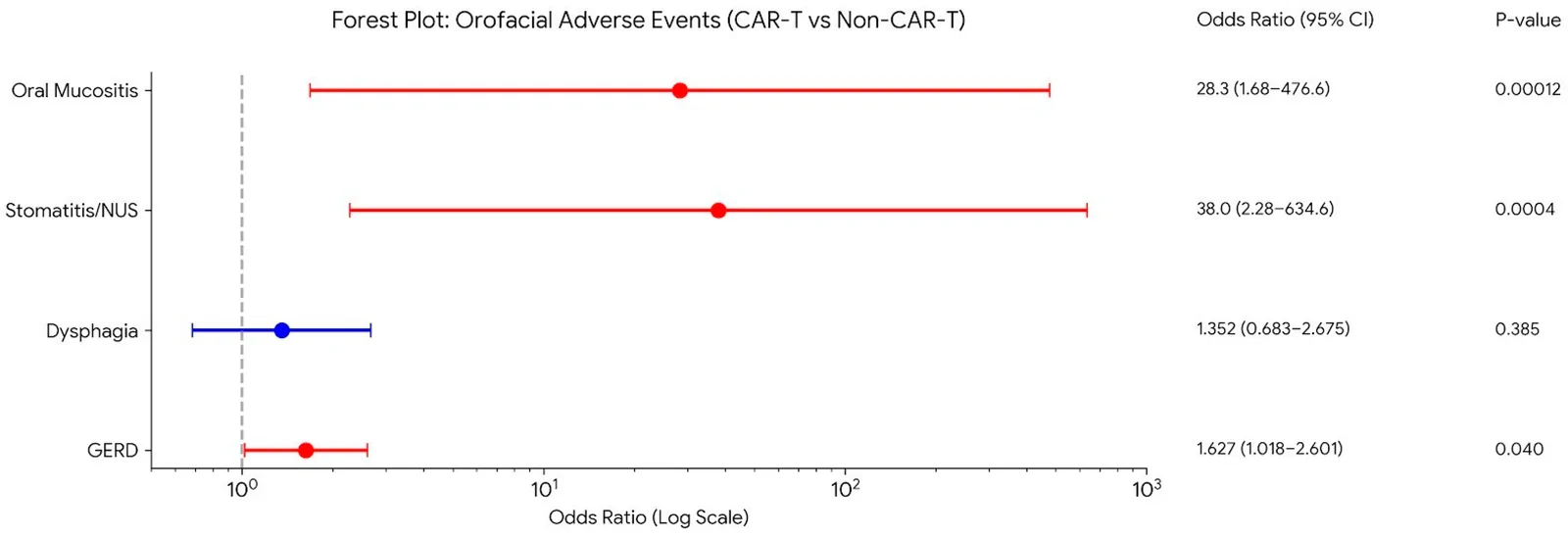
Forest plot showing the magnitude of association of orofacial adverse events between patients with CAR-T therapy and non-CAR-T general population. Red lines depict significance and blue line depict non-significant.

Compared with the general population, CAR-T recipients demonstrated significantly elevated risks for several oral mucosal ulcerative disorders. Oral mucositis was associated with a markedly increased risk (risk ratio of 27.9, *p* = 0.00012), and stomatitis likewise showed a large increase (risk ratio of 37.4, *p* = 0.0004). GERD was modestly but significantly more common among CAR-T patients (risk ratio of 1.60, *p* = 0.04). Dysphagia did not differ significantly from the general population (risk ratio of 1.35, *p* = 0.385). Other orofacial conditions could not be analyzed because event counts (<10) were below the reporting threshold (see Table 3 for effect estimates). Figure 1 is a forest plot showing the magnitude of association of orofacial adverse events between patients with CAR-T therapy and the non-CAR-T general population.

### 3.3. Comparative Risk of Orofacial Adverse Events: CAR-T Versus Hematopoietic Stem Cell Transplantation (HSCT)

**Figure 2:**
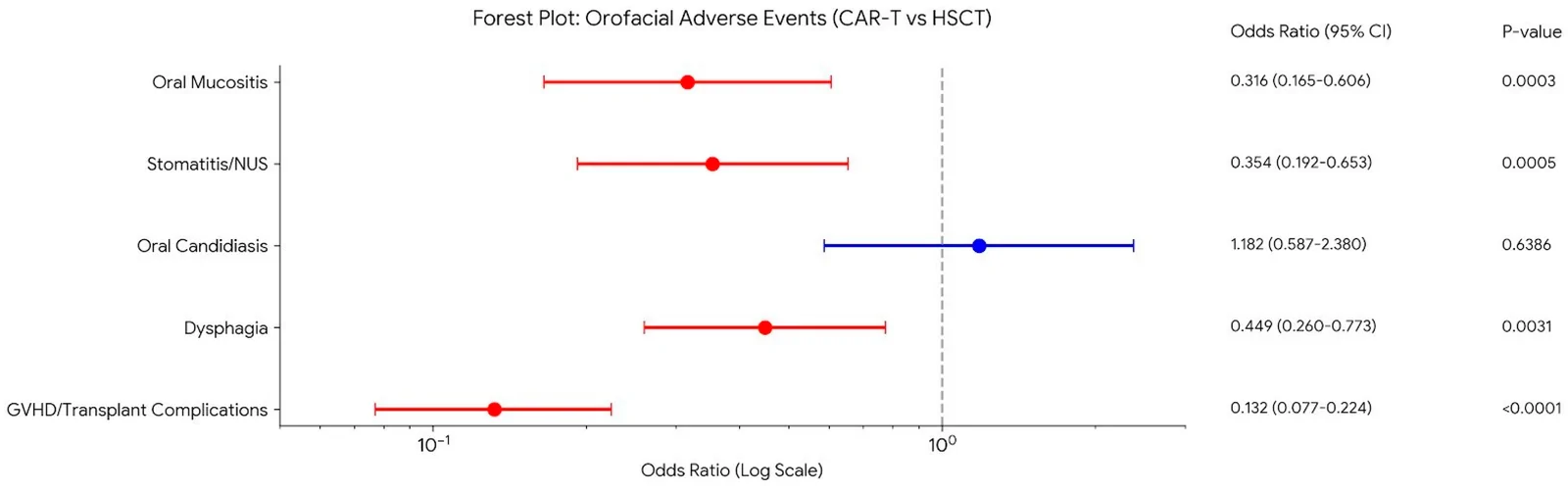
Forest plot showing the magnitude of association of orofacial adverse events between patients with CAR-T therapy and HSCT patients. Red lines depict significance and blue line depict non-significant.

When CAR-T recipients were compared directly with patients who underwent HSCT, CAR-T therapy was associated with a significantly lower risk of several orofacial adverse events. Specifically, CAR-T patients had reduced risks of oral mucositis (risk ratio of 0.324, *p* = 0.0003) and stomatitis (risk ratio of 0.363, *p* = 0.0005) relative to HSCT recipients. Dysphagia was also less frequent after CAR-T (risk ratio of 0.459, *p* = 0.0031), and GvHD was substantially less common in the CAR-T group (risk ratio of 0.145, *p* < 0.0001). Oral candidiasis did not differ significantly between the CAR-T and HSCT cohorts. Remaining orofacial outcomes were either not reported or had counts below the database reporting threshold and therefore were not analyzable (see Table 4 for comparative estimates). Figure 2 is a forest plot showing the magnitude of association of orofacial adverse events between patients with CAR-T therapy and HSCT patients.

## 4. Discussion

The findings extend the limited prior literature by quantifying both absolute incidence and relative risk of orofacial adverse events after CAR-T and by directly contrasting these outcomes with HSCT. CAR-T’s dominant toxicities remain systemic immune syndromes (CRS, ICANS, ICAHT) rather than mucosal or chronic oral complications, a pattern reflected in clinical trials and meta-analyses of CAR-T safety. By contrast, HSCT is consistently associated with high rates of acute mucositis and a substantial burden of graft-versus-host disease/transplant complication, which drive prolonged morbidity and quality-of-life impairment.

Many orofacial adverse events among CAR-T recipients were rare or absent in our cohort. This finding aligns with a small case series by de Paula Eduardo et al. [14], who reported only three treatment-related oral events among ten CAR-T patients: no mucositis or stomatitis, xerostomia in five patients that resolved after discharge, one case of dysgeusia, and one case of oral candidiasis. In our larger sample, the most frequent oral-adjacent adverse event was gastroesophageal reflux disease (GERD; 5.18%), which was significantly more common in CAR-T recipients than in the general population (OR ≈ 1.63). Gastrointestinal (GI) toxicity after CAR-T has been noted elsewhere; for example, Khan et al. [23] reported a high overall frequency of GI adverse events (≈40%), including nausea, vomiting, and diarrhea. The study by Abu-Sbeih et al. also reported the prevalence of GI adverse events for CAR-T therapy as 28% [24]. Oral ulcerative conditions in our cohort, such as mucositis and stomatitis, were uncommon overall (≈2.8% combined), but showed large relative associations versus the general population (ORs ≈ 28.3 and 38.0, respectively). These ulcerative lesions may be related toICAHT, which produces biphasic cytopenias from lymphocyte-depleting conditioning and CAR-T-mediated marrow suppression; neutropenia is a well-recognized risk factor for oral ulceration [25], and may also underlie the cases of oral candidiasis (1.6%) observed here. Although ICANS is a frequent systemic complication of CAR-T, we did not detect a significant association between CAR-T exposure and the orofacial neurologic outcomes evaluated in this study.

Oral adverse events following HSCT are well documented, with reported prevalence estimates approaching 80% [18]. Common adverse events include acute mucositis and stomatitis, xerostomia, oral GVHD, dysgeusia, and opportunistic oral infections [18]. The markedly lower relative risk of oral mucositis, stomatitis, dysphagia, and GVHD/transplant complications after CAR-T versus HSCT likely reflects fundamental differences in treatment biology and conditioning: HSCT patients commonly receive myeloablative chemotherapy and/or total body irradiation and are exposed to alloreactive immune processes that produce GVHD, whereas CAR-T recipients typically do not undergo the same conditioning intensity or allogeneic immune reconstitution. These mechanistic differences plausibly explain the divergent oral toxicity profiles.

Given the low absolute rates observed, routine universal dental interventions solely because of planned CAR-T may not be warranted; however, targeted pre-treatment dental assessment remains prudent, particularly for patients with active dental disease, prolonged neutropenia, or other risk factors for infection. For HSCT candidates, our results reinforce established recommendations for comprehensive pre-transplant dental optimization and long-term orofacial event surveillance due to the high incidence and chronicity of HSCT-related oral complications. A recent clinical practice statement by Multinational Association for Supportive Care in Cancer and International Society of Oral Oncology on dental evaluation and management prior to treatment of patients with CAR T-cell therapy aimed to reduce infections, manage mucosal irritation, and prevent complications during therapy. Key recommendations include enforcing mandatory dental evaluations, prioritizing the elimination of infection sources, and implementing specific management strategies for patients undergoing lymphocyte-depleting conditioning [26].

The limitations of study are that it relies on coded EHR data with potential under-ascertainment of subjective symptoms (e.g., dysgeusia and xerostomia) and small-count suppression that limits analysis of rare outcomes. Differences in surveillance intensity, follow-up duration, and baseline oral health between CAR-T and HSCT populations may introduce residual confounding. Our analysis included HSCT recipients without stratification by transplant type. Subgroup analyses comparing autologous versus allogeneic HSCT recipients were not performed. Further limitations were: lack of information regarding severity or grading of oral adverse events, inability to determine timing of onset, absence of detailed treatment-related variables (CAR-T product, conditioning regimen, supportive medications), and possible coding inaccuracies inherent to electronic health record databases. Additionally, published CAR-T oral data remain sparse and heterogeneous, limiting external corroboration.

Prospective, standardized oral assessments embedded in CAR-T and HSCT clinical programs would clarify incidence, timing, and severity of oral events and enable development of evidence-based dental care pathways. Mechanistic studies comparing mucosal immune responses after CAR-T versus HSCT could identify biological drivers of differential oral toxicity and inform targeted preventive strategies.

## 5. Conclusions

CAR-T therapy carries a distinct and generally lower orofacial toxicity burden than HSCT, but targeted dental assessment and prospective surveillance remain important to optimize supportive care for cellular therapy recipients.

## Figures and Tables

**Table 1:** Treatment and International Classification of Diseases, 10th Revision (ICD-10) codes for CAR T-cell therapy and hemopoietic stem cell transplant patients.

Code Type	Code	Name
RxNorm	1986438	Tisagenlecleucel
RxNorm	1987398	Axicabtagene ciloleucel
RxNorm	2387277	Brexucabtagene autoleucel
RxNorm	2479136	Lisocabtagene maraleucel
RxNorm	2594775	Ciltacabtagene autoleucel
RxNorm	2536430	Idecabtagene vicleucel
RxNorm	2698195	Obecabtagene autoleucel
ICD-10	Z94.81	Bone marrow transplant status
ICD-10	Z48.290	Encountered for aftercare following bone marrow transplant
ICD-10	M31.11	Hematopoietic stem cell transplantation-associated thrombotic microangiopathy

**Table 2:** Prevalence/risk of orofacial adverse events in patients managed with CAR T-cell therapy.

Orofacial Adverse Events	Patients in Cohort *	Patients with Orofacial Adverse Events	Prevalence/Risk (%)
Xerostomia	NR	X	-
Lichenoid Drug Reaction/Drug Eruption	1066	11	1.03%
Oral Mucositis (Ulcerative)	996	13	1.31%
Stomatitis/Oral Ulcerative Disease	985	15	1.52%
Erythema Multiforme	1114	0	0%
Stevens–Johnson Syndrome	1114	0	0%
Oral Lichen Planus	1111	0	0%
Oral Candidiasis	1062	17	1.60%
Oral Leukoplakia	1114	0	0%
Oral Hairy Leukoplakia	1114	0	0%
Gingival Hyperplasia	1113	0	0%
Dysgeusia/Smell–Taste Disturbance	X	X	-
Aphagia	1135	0	0%
Dysphagia	1053	19	1.80%
Neuralgia/Paresthesia-Related Symptoms	NR	X	-
Burning Mouth Syndrome (Glossodynia)	NR	X	-
MRONJ (Osteonecrosis Due to Drugs, Jaw)	1127	0	0%
Dental Caries	NR	X	-
GERD	868	45	5.18%
Dental Erosion	1142	0	0%
Halitosis	NR	X	-
Chronic Pharyngitis/Throat Pain	NR	X	-
Gingival Hypertrophy/Enlargement	1140	0	0%
Graft-Versus-Host Disease (GVHD)-Related Outcomes	NR	X	-
Oral Hyperpigmentation	NR	X	-

*—Cohorts excluded individuals with pre-existing orofacial conditions. X—Precise number unknown; counts of less than 10 are not provided from TriNetX database. NR—Not reported.

**Table 3:** Magnitude of association of orofacial adverse events among CAR T-cell therapy patients.

Orofacial Adverse Events	Cohort	Patients in Cohort *	Patients with Events from Cohort	Risk	*p*-Value	Odds Ratio (95% CI)
Oral Mucositis	CAR-T	995	12	1.21%	0.00012	28.3 (1.68–476.6)
	Non-CAR-T	1112	0	0%		
Stomatitis/NUS	CAR-T	987	16	1.62%	0.0004	38 (2.28–634.6)
	Non-CAR-T	1119	0	0%		
Dysphagia	CAR-T	1029	19	1.85%	0.385	1.352 (0.683–2.675)
	Non-CAR-T	1093	15	1.37%		
GERD	CAR-T	859	42	4.89%	0.040	1.627 (1.018–2.601
	Non-CAR-T	1045	32	3.06%		

*—The analytic cohort consisted of individuals retained after 1:1 age- and sex-matched propensity score matching, comparing CAR-T patients with a control cohort of non-CAR-T, non-chemotherapy, non-radiotherapy, and non-stem cell transplant patients. Both cohorts excluded individuals with pre-existing orofacial conditions. The remaining orofacial adverse events had counts of less than 10 and as such were not reported according to TriNetX HIPPA policy, so no analysis could be performed.

**Table 4:** Magnitude of association of orofacial adverse outcomes between CAR T-cell therapy and hemopoietic stem cell transplant patients.

Orofacial Adverse Events	Cohort	Patients in Cohort *	Patients with Outcome from Cohort	Risk	*p*-Value	Odds Ratio (95% CI)
Oral Mucositis	CAR-T	995	12	1.21%	0.0003	0.316 (0.165–0.606)
	HSCT	1075	40	3.72%		
Stomatitis/NUS	CAR-T	984	14	1.42%	0.0005	0.354 (0.192–0.653)
	HSCT	1073	42	3.91%		
Stevens–Johnson Syndrome	CAR-T	1111	0	0%	1	1(0.02–50.2)
	HSCT	1111	0	0%		
Oral Candidiasis	CAR-T	1059	17	1.61%	0.6386	1.182 (0.587–2.380)
	HSCT	1102	15	1.36%		
Oral Hairy Leukoplakia	CAR-T	1111	0	0%	1	1(0.02–50.2)
	HSCT	1111	0	0%		
Gingival Hyperplasia	CAR-T	1110	0	0%	1	1(0.02–50.2)
	HSCT	1110	0	0%		
Dysphagia	CAR-T	1027	19	1.85%	0.0031	0.449 (0.260–0.773)
	HSCT	1091	44	4.03%		
Graft-versus-host Disease/Transplant Complications	CAR-T	1073	16	1.49%	<0.0001	0.132 (0.077–0.224)
	HSCT	1038	107	10.31%		

*—The analytic cohort consisted of individuals retained after 1:1 age- and sex-matched propensity score matching, comparing CAR-T patients with HSCT patients. Both cohorts excluded individuals with pre-existing orofacial conditions. The remaining orofacial adverse events had counts less than 10 and as such were not reported according to TriNetX HIPPA policy, so no analysis could be performed.

## Data Availability

The data for this study are available upon reasonable request from the corresponding author.
